# Minimally Invasive Internal Fixation of Femoral Shaft Fractures—A Biomechanical Study with a Disruptive Technique

**DOI:** 10.3390/life11111254

**Published:** 2021-11-17

**Authors:** Frank Layher, Georg Matziolis, Leos N. Kayhan, Matthias Bungartz, Olaf Brinkmann

**Affiliations:** 1 Orthopaedic Department Waldkliniken Eisenberg, Orthopaedic Professorship of the University Hospital Jena, 07607 Eisenberg, Germany; f.layher@waldkliniken-eisenberg.de (F.L.); g.matziolis@waldkliniken-eisenberg.de (G.M.); m.bungartz@waldkliniken-eisenberg.de (M.B.); 2 Schulthess Clinic, Lengghalde 2, 8008 Zürich, Switzerland; kayhanll@gmail.com

**Keywords:** femur fracture, biomechanical study, sawbone, internal fixator, disruptive technique

## Abstract

(1) Background: In polytrauma patients, femur fractures are usually stabilised by external fixation for damage control, later being treated with definitive plate or nail osteosynthesis. Screw/rod systems established in spinal surgery might be inserted for internal fixation, providing sufficient fracture stability that subsequent intervention is unnecessary. This was to be investigated biomechanically. (2) Methods: The unilaterally applied spinal internal fixator (IF) was subjected to load and deformation analysis on artificial femurs with 32-A3 fracture according to AO classification. Distance of screws to fracture and rod to cortical bone were analysed as parameters influenced surgically as stiffness and deformation of the treated fracture. In addition, the stability of another construct with a second screw/rod system was determined. The axial load in stance phase during walking was simulated. The results were compared against an established fixed-angle plate osteosynthesis (IP). (3) Results: There were no implant failures in the form of fractures, avulsions or deformations. All unilateral IF combinations were inferior to IP in terms of stability and stiffness. The bilateral construct with two screw/rod systems achieved biomechanical properties comparable to IP. 4) Conclusion: Biomechanically, a biplanar screw/rod system is suitable for definitive fracture stabilisation of the femur, despite a damage control approach.

## 1. Introduction

When treating polytrauma, the patient’s general status requires special consideration. A two-stage procedure with primary damage control and definitive treatment after stabilisation of the patient is designed to improve survival [1]. Plate fixators used for this purpose to date are anatomically shaped and can, therefore, only be inserted in one position. Depending on the soft tissue trauma and/or fracture pattern, this is problematic. Screw/rod systems used in spinal surgery are also internal fixators, but can be applied in any position due to their shape and pliability. This might enable definitive treatment in many cases, despite a damage control approach, in which plate systems appear unsuitable and require a two-stage procedure.

The objective of the present study was to test the hypothesis that an internal fixator (IF) with a screw/rod system used in spinal surgery enables stable osteosynthesis (OS) of a femoral shaft fracture comparable to that achieved with an internal plate fixator (IP; OS plate). At the same time, the optimum application of the screw/rod system was to be determined in order to find the biomechanically most stable arrangement.

## 2. Materials and Methods

The tests were performed exclusively on artificial bones (left femur, 4th gen., composite, 17 PCF solid foam core, size: medium, model 3403) produced by Sawbones (Pacific Research Company, Vashon, WA, USA).

A non-displaced fracture (AO classification 32-A3; extra-articular, transverse femoral shaft fracture, angle <30°, without dislocation at the level of the 2nd–5th sixth of the femur [2]) was produced as a fracture model, using a metal hacksaw and file, with a fracture gap of 2 mm.

To test the alternative fixation method, the spinal fixation system CD Horizon^TM^ Solera^TM^ Spinal System produced by Medtronic GmbH (Meerbusch, Germany) consisting of a rod (cobalt-chrome alloy; Ø 5.5 mm/6.0 mm) and 4 screws (titanium alloy) with 4 polyaxial heads (cobalt-chrome alloy) were used. Pedicle screws from spinal surgery were used for two reasons: (1) it is a proven, matched system; (2) there is currently no better fitting screw-rod connection that could have been considered for the study question. The polyaxial design of the screw heads offers significant advantages for practical application. It simplifies the insertion of the rod system, especially in minimally invasive procedures.

**Figure 1:**
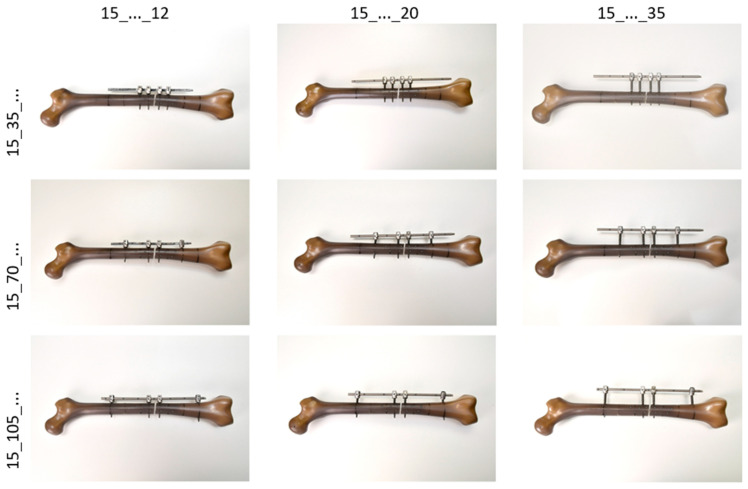
Overview of all variations of the pin2 and rod distances; the column-line labelling indicates the corresponding field in the 3 × 3 test matrix according to the following syntax: Pin1(distance)_Pin2(distance)_Rod(distance).

It was to be expected that the biomechanical stability of the fracture fixation using this screw/rod system (IF) would depend on both the distance of the screws (pins) to the fracture gap and the distance of the rod to the lateral cortex (see Figure 1). The screw position close to the fracture (Pin1) at a distance of 15 mm from the fracture was left unchanged for all variations. The distances of the screw position remote from the fracture (Pin2) varied in three steps of 35 mm each (35 mm, 70 mm, 105 mm). The three rod distances were to be implemented in steps of 15 mm each, starting at 5 mm. However, due to the design of the screw heads, the smallest distance to the bone surface was 12 mm, so that the tests were carried out with rod distances of 12 mm (periosteal), 20 mm (intramuscular) and 35 mm (subcutaneous).

**Figure 2:**
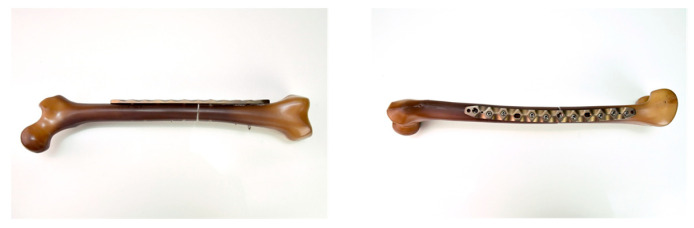
Artificial bone prepared using an osteosynthesis plate (NCB^®^ femur shaft plate) as an IP reference object.

The reference specimen (see Figure 2) was an artificial bone prepared in the same way and treated with an internal plate fixator (IP; NCB^®^ femur shaft plate, curved, 12 holes, 25 mm; Zimmer GmbH, Winterthur, Switzerland). This osteosynthesis plate is made of a titanium alloy (Protasul^®^-64).

The mechanical testing was carried out on a servo-hydraulic testing machine, type Instron 8874 (Instron Germany GmbH, Darmstadt, Germany). The measurement data were obtained with a load cell for combined translational compression (max. force = 10 kN) and rotational torsional loading (max. torque = 100 Nm), with an accuracy of 0.5%. The machine was operated and measured values processed using the software FTStartUp V. 7.22 and MAX V. 9.2 (Instron Germany GmbH, Darmstadt, Germany).

**Figure 3:**
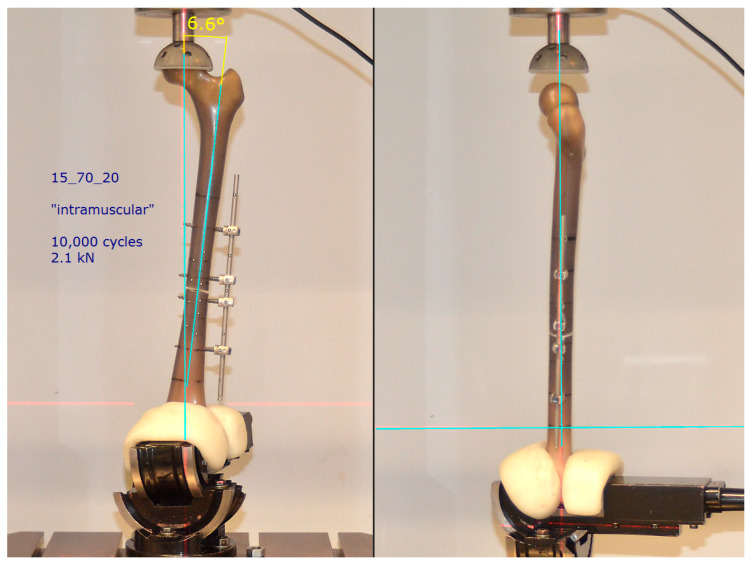
Clamping of the prepared Sawbone (here specimen 15_70_20) in a 6.6° valgus position (frontal) and 0° inclination in sagittal view (adjustment with the aid of a laser plummet).

The measurement situation (specimen arrangement, load size and type) was designed to reflect the true physiological load situation as closely as possible. Accordingly, the following measurement parameters were chosen: With the aid of an industrial vice that can be swivelled around three axes, the femoral shaft was aligned frontally in an anatomical valgus position (approx. 7°). The head was able to rotate without constraint in a cup lined with plastic (artificial acetabular implant), with the centre of rotation in the translation/rotation axis of the testing machine. In the sagittal plane, the perpendicular line ran from the centre of the head through the longitudinal axis of the femur (Figure 3).

The specimens were subjected to an upward and downward swelling load limited to 10,000 cycles. The signal type “walk” was selected as the load pattern, which simulates the vertical, double-peaked load when walking during the stance phase. Based on investigations by the Julius Wolff Institute (Charité Berlin, Germany) [3,4], the maximum load was set to 2.1 kN. This corresponds to approximately 3 times the body weight of a 70 kg individual during normal walking. Each load cycle (1.6 s) was recorded with 100 measurement points. The measured variables time t (s), force F (kN) and distance d (mm) were recorded at each measuring point. As the criterion for assessing the stability of fracture treatment, stiffness was calculated from the ratio of change in force to change in displacement for each cycle.

**Figure 4:**
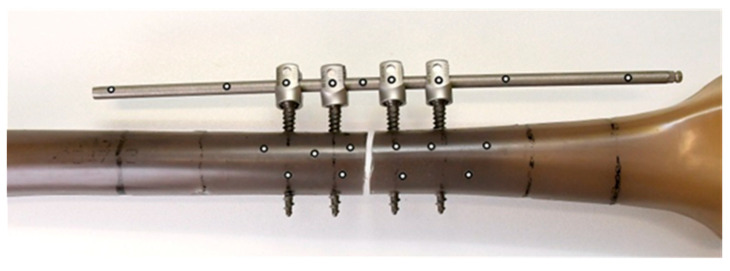
Arrangement of the reference points for ARAMIS measurement (here specimen 15_35_20).

For deformation analysis (compression of the fracture gap, bending), the non-contact optical 3D measuring system ARAMIS (GOM, Braunschweig, Germany) was used as a further measuring method. For this purpose, reference points were attached to the femoral fragments proximal and distal to the fracture site and to the IF/IP implant system (Figure 4). The spatial coordinates of these points were calculated at each measurement point, which made it possible to track the movements of all components.

The spatial coordinates were used to calculate the absolute distances as well as the changes in distance between the proximal and the distal femoral fragment (fracture gap) as well as the bending of the implant as an angle (phi_Femprox_Femdist (°)) and as the change in angle (delta_phi (°)).

## 3. Results

The planned test duration of 10,000 cycles was achieved on all specimens. Neither screw avulsion, implant or bone fractures nor implant failures in the form of plastic deformations were observed. Both the shape of the cyclic loading curves and the shape of the hysteresis changed minimally for all specimens throughout the trial.

**Figure 5:**
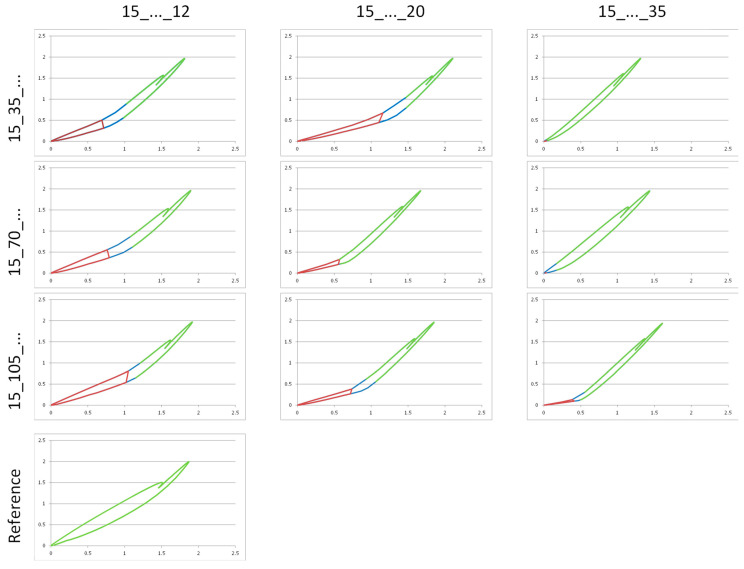
Overview of the curves of the force-displacement measurements of all IF positions for cycle 10,000, compared with IP; two-part hysteresis curve in 3 × 3 test matrix; area with flat increase (red) tends to become smaller with increasing rod distance.

In contrast to IP, the force-displacement hysteresis of all IF combinations did not show a continuous curve (Figure 5). Two phases could be distinguished as a result of different load transfer:

- Phase 1 = Partial hysteresis with a flat increase (low primary stiffness); force transfer by IF alone with the fracture gap still open.
- Phase 2 = Partial hysteresis with a steep increase (high secondary stiffness); combined force transfer by IF and by the bony support of the distal and proximal fracture fragments with a closed fracture gap.

In phase 1, the entire load in the fracture is diverted via the bolt/rod system. If the deformation resistance of the IF combinations decreased due to an increasing rod distance, this had a negative effect on the system stiffness. Phase 1 shortened, the mechanical stability (Stiff_1) of the Pin2-rod arrangements decreased. An ever decreasing force was needed to close the fracture gap. Consequently, the highest stiffness values for Stiff_1 were recorded at the smallest rod distance (12 mm), at approx. 0.6 kN/mm. In the “subcutaneous” situation, this value dropped to approx. 1/3 of the “periosteal” amount. An influence of the screw positions remote from the fracture on the stiffness was not evident. In phase 2, the bony support caused a much higher secondary stiffness level (Stiff_2) of 1.6 kN/mm, which was almost uniform for all IF variants (cf. Table 1).

The test of IP (reference) showed no angled hysteresis in the force-displacement diagram (cf. Figure 5). The implant was, therefore, able to take the maximum load of 2.1 kN completely (no closing of the fracture gap, no phase 2). The stiffness reference value of IP was 1.07 kN/mm, far above that of all IF variants.

The rod distances close to the bone showed comparable results to IP, regardless of the Pin2 positions. However, these almost identical results of the positioning close to the bone and the reference specimen obscure the fact that two different loading forms were present:

- IF system with 2-phase loading—the oscillation amplitudes only record phase 1 of the cycle until contact of the fracture fragments, then no further compression;
- IP system with 1-phase loading—the oscillation amplitudes record the purely elastic deformation of the OS plate over the entire cycle.

**Figure 6:**
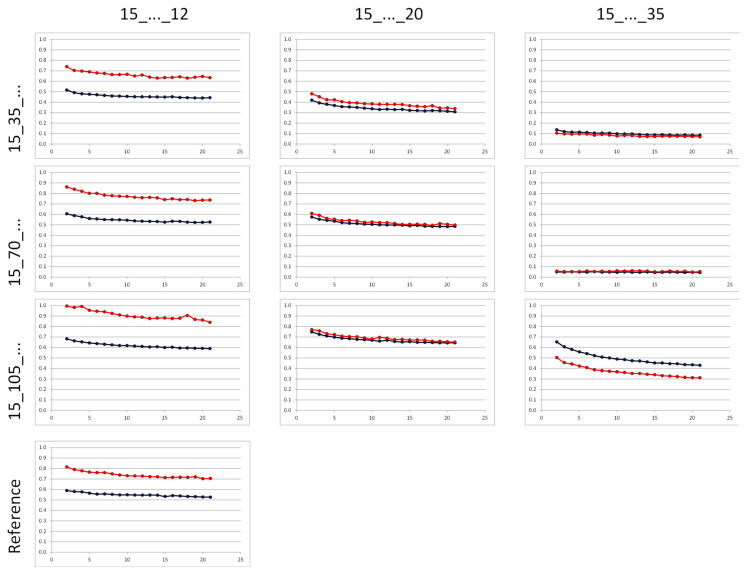
Overview of the curves of distance (blue curve, values in [mm]) and angle differences (red curve, values in [°]) between the proximal and distal femoral part of all IF combinations of the 3 × 3 test matrix in comparison to IP; level of the measured values for the rod arrangements close to the bone is comparable to that for IP (…_12).

Analogous to the stiffness measurement, the influence of the bar spacing was also evident here: the distance and angle differences tended to decrease strongly with increasing bar spacing (Figure 6).

The results of the stiffness and deformation analysis demonstrate that, from a mechanical point of view, none of the IF arrangements tested represent an alternative to the standard treatment (plate osteosynthesis). The stiffness of the IF systems was not sufficient to provide adequate deformation resistance to the load. The applied load resulted in cyclic contact of the distal and proximal fracture fragments.

**Figure 7:**
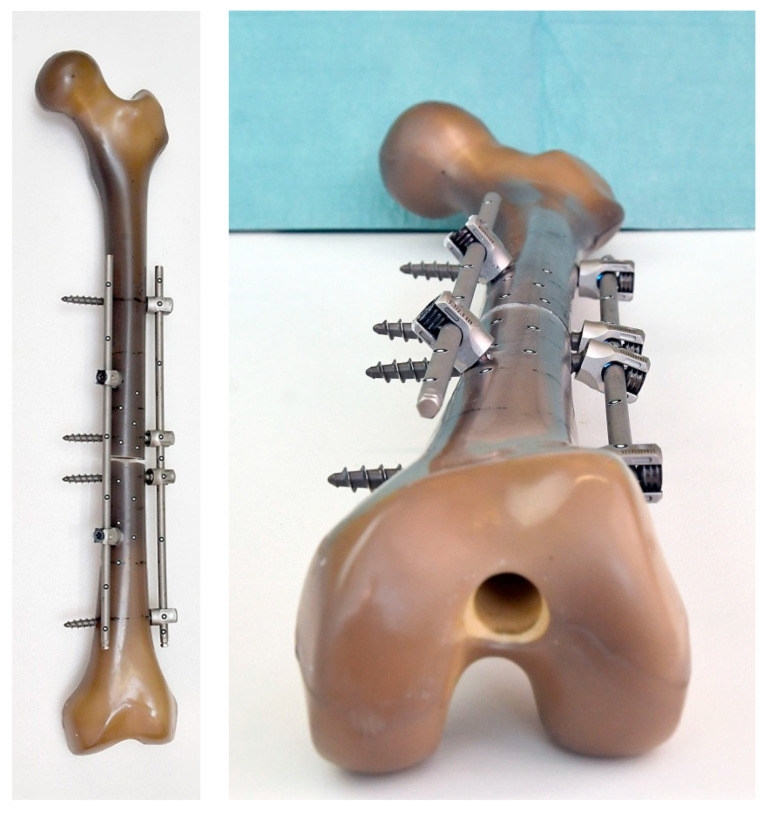
Specimen with additional medial stabilisation based on the IF arrangement with the greatest stiffness in Phase 1.

Based on these results, an additional experiment was conducted. Corresponding to the specimen with the highest stiffness level in phase 1, a Sawbone was provided with additional medial stabilisation (mS). At an angle of 120° around the longitudinal axis of the femur, opposite the lateral IF, an identical rod was again attached close to the bone (12 mm distance) with just two additional screws (screw length 35 mm; screw diameter d = 5.5 mm). The distance of both screws to the fracture site was 50 mm (Figure 7).

The test conditions corresponded to the previous experiments. It was found that the additional medial stabilisation resulted in a substantial increase in stiffness, which was comparable to the reference specimen (1.14 ± 0.06 kN/mm vs. 1.04 ± 0.04 kN/mm, n.s.). Analogously to the OS plate, the fracture gap remained open during the entire loading cycle.

**Figure 8:**
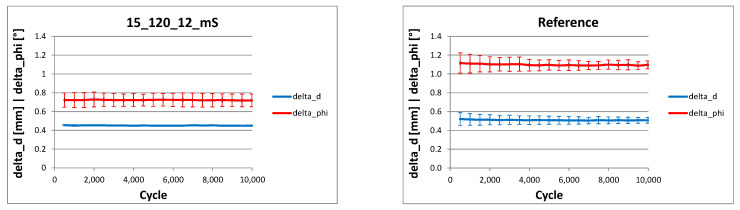
Analysis of the deformation (compression delta_d, bending delta_phi) of specimen 15_105_12_mS and reference specimen IP as a result of the cyclic application of force; mean value as continuous line, standard deviations as error bars.

The deformation analysis also showed comparable results between the two specimens (Figure 8). No significant difference was found between the changes in fracture gap delta_d of specimen 15_105_12_mS (0.45 ± 0.003 mm) and OS plate (0.51 ± 0.04 mm). However, the deformation angle delta_phi between the proximal and distal femoral fragment of specimen 15_105_12_mS (0.72 ± 0.07°) versus IP (1.10 ± 0.06°) was statistically significantly smaller (*p* = 0.005). With comparable compression, the bending was thus markedly restricted.

In order to achieve a stable fracture treatment from a biomechanical point of view, the study results indicate the following requirements for a possible application of the spinal fixation system:

- Fixation of the rod close to the bone (12 mm distance to the cortical bone).
- Proximal and distal outer screws as far away from the fracture as possible.
- Application of additional medial stabilisation, consisting of two screws and a rod also attached close to the bone.

## 4. Discussion

The main result of the present study is that, from a purely biomechanical point of view, a screw/rod system originally used in spinal surgery is suitable for the definitive treatment of femoral fractures, despite a damage control approach, provided that the anchorage rules determined in this study are observed. The clinical relevance of this novel fixation method is to provide a fast, safe and definitive treatment for polytraumatized patients in the so-called damage control phase. To date, a change of procedure has been necessary in many cases during the course of treatment, which represents an additional burden for the patient. In addition, possible complications due following interventions could be avoided (e.g., infection, pain, soft tissue damage due to surgical access). Furthermore, the clinical advantage is that a “closed system” is constructed, thus avoiding the risks associated with an external fixator system.

The stress analysis of this novel fixation method showed appropriate comparative values to the standard plate system used. Based on the measurement data obtained, it can, therefore, be assumed that full loading is possible immediately after fitting with the internal fixator system.

According to the S1 Femur Fracture Guideline [5], the common procedures for the surgical treatment of femoral shaft fractures are intramedullary nail osteosynthesis, plate osteosynthesis and external fixation, with advantages and disadvantages of all three procedures. Depending on the fracture type, soft tissue damage, other severe injuries, previous illnesses and age, these must be selected individually for each patient.

In recent years, closed reduction with minimally invasive, locked intramedullary nail osteosynthesis has become the most common method for the surgical treatment of closed and open diaphyseal femoral fractures of type 32-A1, 32-A2 and 32-A3 [6,7,8,9]. In addition, fixed-angle, polyaxially screwed plate osteosynthesis represents a surgical alternative in the treatment of femoral shaft fractures [10,11,12,13,14]. The third method by means of an external fixator (EF) is used for the rapid temporary stabilisation of fractures of the femoral shaft. It is primarily used in polytrauma, excessive soft tissue damage, extreme vascular or neuronal damage, and thus when no other surgical therapy is initially possible [15,16,17,18].

One-stage definitive treatment of polytraumatised patients is desirable. Internal fixators, as used in spinal surgery, might be an alternative to rigid plate osteosyntheses for the treatment of femoral fractures. To date, however, no comparable biomechanical studies are to be found in the literature.

The present study is the first in which an internal fixator from spinal surgery was investigated for its suitability for the treatment of a femoral fracture. The aim was to find a biomechanically optimal screw/rod arrangement of a spinal rod system for a 32-A3 fracture. The results of stiffness and deformation were referenced against standard treatment with an OS plate (NCB^®^ femur shaft plate).

Numerous factors influence the stiffness of the osteosynthesis and thus also the interfragmentary movement. In order to limit the number of possible influencing parameters, the fracture gap (2 mm) and swing distance (distance of the screws close to the fracture) were maintained for all IF specimens in the study and only one implant system was used. The distance of the osteosynthesis material to the loading axis has a decisive influence on the stability of the fracture treatment. If this distance is increased, the same load may lead to greater deformation [19]. This negative effect on system stiffness was also demonstrated in the present study with increasing rod distance from the bone surface. A single measurement was sufficient to detect this tendency, as the investigations were carried out exclusively on artificial bones (Sawbones, Composite, 4th gen.). Although their mechanical properties (density, strength, hardness, elasticity and fracture resistance) are comparable to human cortical bone [20,21], they have the advantage over donor bone that the properties are virtually constant from specimen to specimen. This high level of reproducibility reduces the scale of the experiment, as an influence of fluctuations in bone properties on the test result can be ruled out. Differences in the test results were thus directly attributable to the influence of the variables screw and rod distance.

Even though the measurement conditions (specimen arrangement, load type and level) were chosen in such a way that they corresponded as far as possible to the physiological load situation during normal walking, the lack of rotational load analysis is to be seen as a limiting factor in this study. As the cyclic force only acted axially, no conclusions could be drawn regarding the stability towards torsional and shear forces. However, in relation to the main axial load, these forces tend to be low in the normal gait cycle [3,4,22]. At the same time, the approach of this study to take the normal load during walking as the maximum axial load (three times the body weight) also includes a large safety margin, since such a high load is not to be expected in trauma patients (inactive, use of walking aids).

For secure fixation of the IF system, all screws must be placed bicortically. In the Sawbone trials, this was easy to check and confirm by visual inspection. In surgical use on patients, only a radiographic check (image intensifier) can ensure safety.

The measurements were limited to 10,000 cycles. From approx. 5000 cycles onwards, the stiffness reached a constant level, so that an extension of the test would not have led to any further gain in knowledge. The lack of screw avulsions and absence of implant or bone fractures also demonstrated the good primary stability of the IF arrangements. Since comparative experiments on donor femurs were not carried out, it is not possible to draw conclusions about transferability to osteoporotic bone conditions, for example. After the principle suitability of this novel fixation method has been proven, further tests in animal models as well as on human donor bones are planned in further studies.

## 5. Conclusions

The results show that none of the unilateral IF combinations provided fracture stabilisation comparable to that of the IP specimen. Only by attaching a further rod, using just two additional screws inserted medially, were the reference stiffness level and a comparable deformation achieved. According to [23], stable fixation should still be flexible enough to initiate secondary fracture healing. The results of approx. 1.14 kN/mm (IF) and 1.04 kN/mm (IP) for axial loading are, in accordance with [24], within an optimal stiffness range that promotes the healing process (1.0–2.5 kN/mm). It is precisely in this moderate range that the callus exhibits high strength and stiffness [25].

The choice of materials might be a matter for further investigations. The strength of the rods or their material properties can influence the stability of the overall construct.

In addition, the most atraumatic surgical approach for the application of two screw/rod systems in the manner determined here should be established in follow-up anatomical investigations, taking into account “safe zones” [26].

## Figures and Tables

**Table 1:** Stiffness values of phase 1 (Stiff_1) and phase 2 (Stiff_2) [kN/mm] determined by regression, depending on pin2 and bar distance (3 × 3 test matrix) in cycle 10,000.

Stiff_1	Rod Distance			Stiff_2	Rod Distance		
Pin2 distance	12 mm	20 mm	35 mm	Pin2 distance	12 mm	20 mm	35 mm
35 mm	0.58 kN/mm	0.49 kN/mm	0.26 kN/mm	35 mm	1.57 kN/mm	1.67 kN/mm	1.57 kN/mm
70 mm	0.60 kN/mm	0.48 kN/mm	0.24 kN/mm	70 mm	1.53 kN/mm	1.63 kN/mm	1.52 kN/mm
105 mm	0.65 kN/mm	0.45 kN/mm	0.29 kN/mm	105 mm	1.60 kN/mm	1.61 kN/mm	1.65 kN/mm
